# A Novel Aza-Derivative Inhibits *agr* Quorum Sensing Signaling and Synergizes Methicillin-Resistant *Staphylococcus aureus* to Clindamycin

**DOI:** 10.3389/fmicb.2021.610859

**Published:** 2021-02-09

**Authors:** Giulia Bernabè, Matteo Dal Pra, Vittoria Ronca, Anthony Pauletto, Giovanni Marzaro, Francesca Saluzzo, Annalisa Stefani, Ilaria Artusi, Vincenzo De Filippis, Maria Grazia Ferlin, Paola Brun, Ignazio Castagliuolo

**Affiliations:** ^1^Department of Molecular Medicine, University of Padua, Padua, Italy; ^2^Department of Pharmaceutical and Pharmacological Sciences, University of Padua, Padua, Italy; ^3^Azienda Ospedale Università di Padova, Padua, Italy; ^4^Istituto Zooprofilattico Sperimentale delle Venezie, Legnaro, Italy

**Keywords:** MRSA, antibiotic resistance, quorum sensing, quorum sensing inhibitor, anti-virulence compound

## Abstract

Increasing antibiotic resistance and diminishing pharmaceutical industry investments have increased the need for molecules that can treat infections caused by dangerous pathogens such as methicillin-resistant *Staphylococcus aureus* (MRSA). Quorum Sensing (QS) is a signaling mechanism that regulates bacterial virulence in pathogens. A report demonstrating that the anti-inflammatory drug Diflunisal reduces MRSA virulence factors’ expression prompted us to design, synthesize and test 16 aza-analogs as inhibitors of *S. aureus* virulence factors controlled by the accessory gene regulator (*agr*) QS system. At first, we evaluated by qRT-PCR the activity of compounds on *rnaIII* expression, a QS related gene. Azan-7 was the most active molecule tested and it did not show cytotoxic activity in human cell lines. Moreover, we demonstrated that it did not affect bacterial proliferation. Regulation of MRSA virulence genes by Azan-7 was investigated using qRT-PCR and RNAseq. Azan-7 significantly reduced *hla*, *psm*α, *hysA*, *agrA*, *cap1A*, and *cap1C* gene expression. *In silico* docking demonstrated that Azan-7 binds the response regulator AgrA. This data was confirmed by electrophoretic mobility shift assay (EMSA) reporting that Azan-7 binding to AgrA protein strongly reduced the AgrA-DNA complex formation at the P3 promoter region involved in the regulation of *rnaIII* transcription. Azan-7 inhibited MRSA-mediated haemolysis, reduced survival of the pathogen at low pH levels, and increased macrophage killing. In addition, Azan-7 enhanced MRSA susceptibility to clindamycin both in planktonic growth and biofilm. Azan-7 did not induce resistance over 10 days in culture. It was equally active against all the AgrA MRSA subtypes encountered among clinical isolates, but it was not active against *Staphylococcus epidermidis*, although the AgrA proteins show an approximate 80% homology. These results demonstrate that Azan-7 inhibits the expression of MRSA virulence factors by interfering in the QS and synergizes MRSA biofilm with clindamycin, indicating the compound as a promising candidate for the treatment of MRSA infections.

## Introduction

Antibiotic resistance has become a global public health problem, and antibiotic-resistant pathogens pose a growing risk and burden for human health. The efforts to eradicate antibiotic resistance and develop new drugs to treat resistant bacteria imply costly and complicated solutions to meet the global challenge (Laxminarayan et al., 2013). Antibiotic resistance occurs when bacteria survive the effects of an antimicrobial drug that was initially effective in treating the infection (Medina and Pieper, 2016). The organizations that monitor human infections, including the Centers for Disease Control and Prevention (CDC), the National Institutes of Health (NIH), the European Centre for Disease Prevention and Control (ECDC), and the World Economic Forum, have been alerting health officials about the dangerous diffusion of resistant strains and the rapid reduction in the efficacy of antibiotics due to resistance mechanisms and errors in drug administration (Spellberg et al., 2016). It has been estimated that by 2050 infectious diseases caused by resistant pathogens will affect 10 million lives per year and account for 100 trillion dollars of global economic losses (O’Neill, 2016).

*Staphylococcus aureus*, one of the most widespread pathogens in the community and hospital-acquired infections (Tong et al., 2015), shows an extraordinary ability to acquire resistance to newly developed antibiotics (Deleo et al., 2009). Although *S. aureus* is known to be part of the normal skin microbiota, it is frequently the cause of infections involving the respiratory tract, the skin, the soft tissues, and the bloodstream (Reddy et al., 2017). *S. aureus*-related infections have become more challenging to treat over recent years due to an increasing prevalence of multi-resistant strains. Indeed, in 2017, 16.9% of hospital-acquired infections in Europe were caused by methicillin-resistant *S. aureus* (MRSA) (Centre for Disease Control and Prevention, 2013; European Centre for Disease Prevention and Control, 2018).

The severity of bacterial infections depends on the virulence of the specific strain (Cheung et al., 2011). The virulence of *S. aureus* partially relies on its Quorum Sensing (QS) system, which enables the pathogen to adapt to the environment rapidly and coordinate gene expression in the bacterial population (Yarwood and Schlievert, 2003; Bien et al., 2011). The accessory gene regulator (*agr*) locus is a master regulator in the QS-system of *S. aureus* and consists of two divergent operons driven, respectively, by the P2 and P3 promoters. The P2 operon controls the expression of AgrB and AgrD, involved in the processing of autoinducing peptide (AIP), and of AgrC and AgrA proteins that respond to extracellular AIP (Gong et al., 2014). The P3 operon regulates RNAIII expression that in turn controls gene-related switch from an adhesive, biofilm formation to an invasive, toxic phenotype of the pathogen. AIP activates AgrC, which can then phosphorylate AgrA that acts as a transcription factor to facilitate the transcription of RNAII and RNAIII. Concentration of AIP rises with bacterial density, and as the density of the colony increases, this feedback loop leads to enhanced AIP expression and activity (Yarwood and Schlievert, 2003).

Given the rising rates of antibiotic resistance, QS inhibitors may provide an alternative to conventional antibiotic therapy. Recently, it has been reported that diflunisal, an FDA-approved non-steroidal anti-inflammatory drug, inhibits AgrA protein binding to the oligonucleotide corresponding to the DNA sequence of the P3 promoter, disrupting the QS signal in *S. aureus* (Khodaverdian et al., 2013). A ligand-binding pocket on the C-terminal DNA-binding domain of AgrA (Koenig et al., 2004) has recently been identified, raising the hypothesis that diflunisal binds to the C-terminal domain, or perhaps to both AgrA domains. The current study set out to examine some aza-analogs in the effort to identify a molecule that is devoid of significant toxic effects yet capable of inhibiting the binding of AgrA to P3 promoter and of playing a robust anti-virulence role against MRSA. Host defense could be supported by inhibiting bacterial QS signaling thus rendering the pathogenic bacteria avirulent and/or less fit for survival in the host.

## Materials and Methods

### Bacterial Strains and Growth Conditions

Methicillin-resistant *S. aureus* strain obtained from ATCC (strain number 33592) was tested. *Staphylococcus epidermidis* and *S. aureus* clinical isolates (*n* = 57) were obtained from the Microbiology Laboratories of University of Padova and University of Urbino and subjected to morphological, biochemical, and molecular typing as described elsewhere (Hall et al., 2013). The strains were grown in Lysogeny broth (LB) (Becton Dickinson).

### Total RNA Isolation and Quantitative RT-PCR

Methicillin-resistant *S. aureus* was cultured for 1–120 h in LB agar alone or supplemented with Diflunisal (Sigma) or aza-analogs previously dissolved in dimethyl sulfoxide (DMSO) (Sigma) (Carta et al., 2018). The bacterial cultures were centrifuged and total RNA was isolated and purified from the bacterial pellets using the GRS Total RNA Kit – Bacteria (#GK16.0100, GRISP Research Solution) following the manufacturer’s instructions. Purified RNA was subjected to DNase I treatment to remove contaminating DNA. The RNA yield and purity were assessed by measuring the absorbance, and only samples with a ratio 260/280 nm in the 1.8–2 range were used. cDNA was generated using cDNA RT kit with an RNAse inhibitor (Applied Biosystems). Quantitative PCR (qRT-PCR) was performed using SYBRR green mixture (iScript One Step RT-PCR kit with SYBR green, Bio-Rad) to determine transcript levels of genes using oligonucleotides listed in Table 1. 16S and *gyrB* genes served as housekeeping genes. The data were analyzed using the ΔΔCt method; the samples incubated with vehicle (DMSO 0.05%) were used as the control. All the samples were assessed in triplicate.

**TABLE 1:** Sequences and annealing temperatures of oligonucleotides used in the qRT-PCR experiments.

Genes	Oligonucleotides	Annealing temp (°C)
*16S*	fw 5′-AAACTCAAAKGAATTGACGG-3′	60°C
	rv 5′-CTCACRRCACGAGCTGAC-3′	
*gyrB*	fw 5′-CAAATGATCACAGCATTTGGTACAG-3′	60°C
	rv 5′-CGGCATCAGTCATAATGACGAT-3′	
*rnaIII S. aureus*	fw 5′-TTCACTGTGTCGATAATCCA-3′	60°C
	rv 5′TGATTTCAATGGCACAAGAT-5′	
*rnaIII S. epidermidis*	fw 5′-ACTAAATCACCGATTGTAGAAATGATAT CT-3′	62°C
	rv 5′-ATTTGCTTAATCTAGTCGAGTGAATG TT A-3′	
agrA	fw 5′-GCACATACACGCTTACAATTGTTG-3′	59°C
	rv 5′-ACACTGAATTACTGCCACGTTTTAAT-3′	
hla	fw 5′ATGGATAGAAAAGCATCCAAACA-3′	62°C
	rv 5′-TTTCCAATTTGTTGAAGTCCAAT-3′	
psmα	fw 5′-TATCAAAAGCTTAATCGAACAATTC-3′	60°C
	rv 5′-CCCCTTCAAATAAGATGTTCATATC-3′	
cap1A	fw 5′-AGGCATGTCATGAGCAAAAACT-3′	62°C
	rv 5′-TGTCTTGTAGTAAGTGCGGCAT-3′	
cap1C	fw 5′-TTGCACATCCAGAGCGGAAT-3′	62°C
	rv 5′-TGCGCATCTGAACCGATGAA-3′	

### Growth Inhibition Assay

An overnight culture of MRSA was collected, centrifuged, and dispensed in 96-well microtiter plates at 1 × 10^6^ CFU/well final concentration. Aza-derivatives or Diflunisal were added to bacterial cultures at final concentrations ranging from 0 to 100 μM. The plates were incubated at 37°C under continuous shaking for 24 h. Bacterial growth was quantified at specified time points by measuring the optical density at 620 nm. All the experiments were repeated at least three times with duplicate determinations for each condition.

### Cytotoxicity Assay

Caco2 (human epithelial colorectal adenocarcinoma cells, ATCC^®^ HTB-37^TM^) and A549 (human alveolar basal epithelial carcinoma cells, ATCC^®^ CCL-185^TM^) cells were seeded in 96 well/plates (1 × 10^4^ cells/well) in Dulbecco’s modified Eagle’s medium (DMEM) supplemented with 20% fetal bovine serum (FBS) and 1% penicillin/streptomycin or RPMI 1640 with 10% FBS and 1% penicillin/streptomycin, respectively. Twenty-four hours later, cells were incubated with Azan-7 concentrations ranging from 0 to 2 mM. Treatment using the highest final DMSO concentration (0.05%) was used as control. After additional 24 h at 37°C, the culture medium was removed, replaced with fresh complete medium, and incubated for 48 h. To assess cell availability, cultures were incubated with MTT (3-(4,5-dimethylthiazol-2-yL)-2,5-diphenyltetrazolium bromide) solution (5 mg/mL) for 4 h at 37°C. Formazan crystals were solubilized in 100 μL of SDS 10% w/vol HCl 0.01 N, and the absorbance was recorded 16 h later at 590 nm using a microplate reader (Sunrise; Tecan, Switzerland).

### Rabbit Red Blood Cells Haemolysis Assay

The haemolytic activity of MRSA was assessed using a functional test that measures the release of hemoglobin from erythrocytes. MRSA (10^6^ CFU/ml) was grown at 37°C in a shaking bath in the absence or presence of the compounds. After 16 h, the cultures were centrifuged (6,000 × g 4°C), and the supernatants were sterile filtered (0.22 μm). 100 μL were added to 900 μl haemolysin buffer (0.145 M NaCl, 0.02 CaCl_2_) and 25 μl of defibrinated rabbit blood. The samples were incubated in an orbital shaker at 37°C for 1 h. Then the samples were centrifuged (5,500 × g, room temperature, 1 min) to pellet intact erythrocytes, and the supernatants were transferred to a 96 well plate to measure absorbance at 541 nm. Sterile culture medium was used as the reference for 0% haemolysis, and a bacterial culture supernatant devoid of any treatment served as the standard for 100% haemolysis. The percentage of haemolysis inhibition was calculated over the control cultures. All the assays were performed at least twice with triplicate determinations for each condition.

### Macrophage Intracellular Killing

Murine macrophage RAW264 cells (kindly provided by Prof. P. Bonaldo, University of Padova) were maintained at 37°C in 5% CO_2_ in high-glucose DMEM supplemented with 10% FBS, 2 mM L-glutamine, 10 mM HEPES, with 100 U/ml penicillin and 100 g/ml streptomycin. For the experiments, MRSA were cultured overnight in LB medium with or without 100 μM Azan-7. RAW264 cells were detached by trypsinization, washed, and suspended at 2 × 10^7^ cells/ml in DMEM with 1% FBS without antibiotics. RAW264 cells were then combined with MRSA at a multiplicity of infection (MOI) 1:1. Samples were centrifuged at 500 × g for 3 min to facilitate cell-bacteria contact and incubated at 37°C in 5% CO_2_ for 1 h to allow phagocytosis. At the end of incubation, samples were treated with lysostaphin (Sigma-Aldrich) (2 mg/ml for 15 min) to kill extracellular bacteria and subsequently centrifuged (1,800 rpm for 8 min). To enumerate phagocyted bacteria, samples were suspended in PBS/0.1% Triton-X-100 to disrupt macrophage membranes and then serially diluted and seeded onto LB agar for 16 h at 37°C. In a parallel set of experiments, samples were resuspended in fresh culture media and incubated for 4 h at 37°C before cell membrane disruption and bacterial enumeration.

### Low pH Killing

Methicillin-resistant S. aureus overnight cultures (10^6^ CFU) were incubated for 16 h in LB supplemented with Azan-7 (100, 50, and 10 μM) at 37°C with shaking. The bacteria were then centrifuged, washed, and resuspended at 10^8^ CFU/ml into a low pH broth (DMEM/2% Hepes, pH 2.5). After 2 h, live bacteria were enumerated by serial dilutions and plating on LB agar.

### Expression and Purification of S. aureus AgrA

Recombinant AgrA was expressed and purified as described elsewhere (Nicod et al., 2014). Expression plasmid pSN-agrA was kindly provided by Prof. Sivaramesh Wigneshweraraj and Lynn Burchell of the Imperial College London (Nicod et al., 2014). Briefly, Escherichia coli strain BL21(DE3)pLysS was grown at 37°C to OD600 ∼0.5. The culture flask was transferred at 20°C and left to equilibrate for 30 min. AgrA expression was induced with 0.5 mM IPTG. After 16 h of incubation at 20°C with shaking, AgrA was purified using the IMPACT kit (New England Biolabs) following the manufacturer’s instructions. Briefly, the culture was centrifuged (4000 × g, 4°C) and the bacterial pellet was resuspended in column buffer (20 mM Tris–HCl pH 9. 1 M NaCl and 1 mM EDTA) and lysed through cycles of sonication and thermal shock for 6 min (30 s on-off cycle). Samples were centrifuged (8,000 × g at 4°C) for 1 h. The clear supernatant was loaded on a gravity flow column previously packed with Chitin Resin (New England Biolabs). Column was washed with five volumes of column resin bed of column buffer and incubated for 16 h at 4°C in cleavage buffer (Column Buffer + 200 mM DTT). The eluted protein was concentrated using Amicon Ultracell-10K (Millipore) and quantified by Pierce^TM^ BCA Protein Assay Kit (Thermo Fisher). The purity of the recombinant protein was confirmed by SDS-Page gel. AgrA was stored in storage buffer (10 mM Tris–HCl pH8, 50 mM NaCl, 0.1 mM EDTA, 1 mm DTT, and 20% glycerol) at −80°C.

### Electrophoretic Mobility Shift Assay (EMSA)

Electrophoretic mobility shift assay (EMSA) was performed following the method described by Khodaverdian et al. (2013). Sequences of P2 and P3 promoter regions (Nikolskaya and Galperin, 2002) and the negative control are reported in Table 2 and were synthesized with a 3′ 6-fluorescein (FAM) (Sigma). Binding reactions involving protein AgrA, DNA probe, vehicle, or Azan-7 were set up in reaction buffer [10 mM HEPES pH 7.6, 2 mM dithiothreitol (DTT), 50 mM KCl, 1 mM EDTA, 0.05 % Triton X-100 and 5% glycerol]. The reaction was carried on at 25°C for 30 min. The assays were run with 5% native polyacrylamide gels in 0.5× Tris-Borate-EDTA (TBE) at 4°C. The gels were visualized and quantified using GE Typhoon FLA 2000 PhosphorImager. Band density was evaluated using ImageQuant TL software.

**TABLE 2:** Sequences of the promoter used in the electrophoretic mobility shift assay.

Name	Sequence
P2	5′ [6FAM]-TACATTTAACAGTTAAGTATTTATTTCCTACAGTTAGG
	CAATATAATG-3′
P3	5′-[6FAM]-AATTTTTCTTAACTAGTCGTTTTTTATTCTTAACTGTAA
	ATTTTT-3′
NegControl	5′-[6FAM]-CCTGGTTGTCCTCGTCACTATGAAGAGCCTCACACAC
	AAGGTCGTCGA-3′

### Evaluation of AgrA-Azan-7 Binding by Fluorescence Spectroscopy

Binding measurements between Azan-7 compound and AgrA were performed by adding to constant substrate concentration (200 nM) increasing amount of AgrA protein (0–700 nM), in 20 mM Tris–HCl, pH 8.0, 50 mM NaCl, 0.1% PEG-8000 (w/v) (TBS). After incubation at 37°C for 15 min, the fluorescence data were collected exciting each sample at 342 nm, corresponding to Azan-7 maximum absorbance, using an excitation/emission slit of 5 and 10 nm. The emission intensity was recorded at 475 nm, Azan-7 λ_max_, after subtracting the corresponding signal of AgrA alone.

The data points were interpolated with the following equation, describing a single-site binding model:

$$y=\frac{\left(F_{0}+F\cdot \left(\frac{x}{K_{\text{d}}}\right)\right)}{\left(1+\left(\frac{x}{K_{\text{d}}}\right)\right)}$$

where *F*_0_ and *F* are the fluorescence intensity of Azan-7 alone and Azan-7 at each AgrA concentration, respectively, and K_d_ is the equilibrium dissociation constant of Azan-7-AgrA complex, obtained as a fitting parameter.

### Computational Methodology

All the computational studies were carried out on a 4 CPU (Intel Core2 Quad CPU Q9550, 2.83 GHz) ACPI x64 Linux workstation with Ubuntu 12.04 operating system. The structure of AgrA LytTR domain was retrieved from the Protein Data Bank (PDB code 4G4K) (Leonard et al., 2012). The structure was submitted to the DockPrep utility of UCSF Chimera (Pettersen et al., 2004), thus replacing missing side chains, adding hydrogen atoms, and computing the partial charges. The structure of Azan-7 was prepared with MarvinSketch 5.5 software^1^, determining the lowest energy conformation and the degree of protonation at pH = 7.2 using the MMFF94s force field. The input molecules were converted in the corresponding “.pdbqt” file format for the next docking studies. Docking simulations were performed with AutoDock 4.2 software (Allouche, 2012) using a grid box of 40 × 40 × 36 dimensions (grid spacing = 0.375 Å) and centered at *x* = 7.80, *y* = 3.02, *z* = 34.11. Fifty runs with a maximum of 2,500,000 energy evaluations were carried out using the Lamarckian Genetic Algorithm (LGA) searching engine. Cluster analysis was performed on the docked results, with a root-mean-square tolerance of 2.0 Å. The lowest energy pose was then refined running another docking simulation in which the LYS236 sidechain was considered flexible.

### RNA-Seq and Gene Expression Analysis

RNA sequencing (RNA-Seq) was performed using an unbiased approach for analyzing and quantifying bacterial transcripts. Thus, 1 × 10^6^ CFU/ml bacteria were grown for 16 h with Azan-7 or vehicle (DMSO 0.05%). The bacterial RNA was purified as described above, and integrity was evaluated by agarose gel electrophoresis and BioAnalyzer.

RNA sequencing libraries were prepared with the TruSeq RNA Sample Prep kit (Illumina, San Diego, CA, United States). The total RNA samples were subjected to poly(A) enrichment, and 100 nucleotides of sequence was determined from both ends of each cDNA fragment using the HiSeq platform (Illumina) following the manufacturer’s protocol. Sequencing reads were annotated and aligned to the USA300 MRSA (Diep, 2006) reference genome using TopHat. Comparisons were made between MRSA treated with vehicle and Azan-7 (100 μM). We selected *a priori* a set of genes known to be functionally related to the *agr* QS system to conduct an in-depth analysis. More specifically, we retrieved all the genes that were used to construct a Boolean network describing QS in *S. aureus* (Audretsch et al., 2013). All the genes included in previous studies are listed in Supplementary Table 1 and were used as an “*a priori*” list to perform searches in the differential expression analysis. The following algorithm was used: if the gene name is available from the *a priori* list, perform a search for the gene name; if the previous step is unsuccessful (or the gene name is not available from an *a priori* list), perform a search using any term of the description. The results were then checked against the UniProt Knowledge base^2^ (reporting both gene name and gene description) to ensure concordance between the gene listed in the *a priori* list and the gene selected by the text search in the DE results. For each comparison, we present the results for the genes included in the work described above and that were significantly over or under-expressed in comparison with the untreated samples.

### *In vitro* Resistance Development

The development of resistance in response to Azan-7 was tested following a method described elsewhere (Sully et al., 2014). Briefly, an overnight culture of MRSA was collected, centrifuged, and resuspended in LB at 1 × 10^6^ CFU/mL. Clindamycin (0.25 μg/mL, Sigma), Azan-7 (100 μM), or vehicle (DMSO 0.05%) were added to 1 mL of culture and incubated at 37°C with shaking. Twenty-four hours later, bacteria were collected by centrifugation, resuspended into 1 mL of fresh LB medium. Antibiotic, compound, or vehicle was renewed. This protocol was repeated for 10 days. At the end of the treatment, bacterial cultures were incubated for 16 h with increasing doses of the antibiotic and then plated on LB agar to count the CFUs. In a parallel set of experiments, total RNA was extracted from bacterial cultures and the expression of RNAIII, *agrA*, and *hla* was determined by qRT-PCR.

### Evaluation of MRSA Biofilm Formation

Overnight cultures of MRSA were diluted 1:100 in fresh LB and incubated at 37°C for 2 h. The bacterial suspension was then diluted 1:1000 in LB broth and 200 μL/well were seeded into 96 well polystyrene microtiter plates and incubated at 37°C under static conditions. Following 16, 24, 48, 72, or 96 h of incubation, the wells were emptied and washed three times with sterile PBS to remove planktonic cells. Adhering cells were incubated with resazurin 0.01% in the dark at 37°C for 20 min (Kirchner et al., 2012). The non-fluorescent resazurin (blue) was reduced to highly fluorescent resorufin (pink) by dehydrogenase enzymes in metabolically active cells. The relative fluorescence units (RFU) of resorufin were measured using a fluorimeter (Ex = 530–570 nm, Em = 590–620 nm) (Perkin Elmer Victor X2 Multilabel Microplate Reader).

### Synergistic Activity of Azan-7 and Clindamycin

To check the synergy of Azan-7 and Clindamycin, checkerboard assay was set up and FIC values were calculated as previously described by Rabadia et al. (2013). MRSA cultures (10^6^ CFU) were added to 96-well plates where clindamycin was serially diluted along the ordinate and Azan-7 was diluted along the abscissa. Plates were incubated for 16 h at 37°C and the optical density was measured to evaluate bacterial growth. The ΣFIC values were calculated as follows:

ΣFIC=FICA+FIC,B

where FIC_A_ is the MIC of drug A (Clindamycin) in the combination/MIC of drug A (Clindamycin) alone, and FIC_B_ is the MIC of drug B (Azan-7) in the combination/MIC of drug B (Azan-7) alone.

**Table:** 

***FIC value***	
*Synergy*	<0.5
*Antagonism*	>4
*Additive or indifference*	0.5–4

### Effect of Azan-7 on Clindamycin Activity in Planktonic Culture and Biofilm Growth

Overnight cultures of MRSA were seeded in 96-well microtiter plates (1 × 10^6^ CFU/well) with Azan-7 (100 μM), Azan-7 (100 μM) plus clindamycin (0.25 μg/mL), or clindamycin (0.25 μg/mL) alone. After 24 h at 37°C with shaking, the growth in planktonic cultures was measured at 620 nm using a microplate reader.

To assess the effect of Azan-7 with or without antibiotic on biofilm growth, MRSA overnight cultures were diluted 1:100 in fresh LB and incubated at 37°C for 2 h. The bacterial suspensions were then diluted 1:1000 in LB broth and 200 μL/well were seeded into 96 well polystyrene microtiter plates and incubated at 37°C under static conditions. Following 36 h of incubation, clindamycin (1 μg/mL) and Azan-7 (100 μM) were added as specified. After an additional 16 h of incubation at 37°C, the LB medium was discarded, each well was washed three times with sterile PBS to remove planktonic cells, and adhering bacteria were stained with 150 μl of 0.1% (w/v) Crystal violet (CV) solution for 15 min at room temperature. The plates were washed, air-dried, and CV was solubilized in 125 μl of 30% (v/v) glacial acetic acid per well. The optical density was measured at 570 nm using a microplate reader (Sunrise; Tecan, Switzerland). Bacteria incubated with DMSO 0.05% assigned 100% biofilm formation. All the experiments were performed at least three times with triplicate determinations for each condition.

### Clinical Isolates

Fifty-seven *S. aureus* isolates were obtained from clinical specimens routinely submitted to the Clinical Microbiology Laboratories of the University Hospitals of Padova and Urbino (Italy) between January 2019 and June 30, 2019. *S. aureus* isolates were identified using conventional laboratory approaches, including Gram staining, colony morphology, MALDI-TOF analysis, and antibiotic susceptibility tests.

### Typing Agr Groups in *S. aureus* Isolates

Overnight culture (1 ml) of *S. aureus* isolates were centrifuged at 3,800 rpm for 10 min. Bacteria were resuspended in 500 μL of Lysis Buffer (NaCl 10 mM, MgCl_2_ 3 mM, Tris–HCl 20 mM pH 7.4, 0.3% Nonidet P40, 1.25% sucrose), 62.5 μL of SDS 10% and 20 μL of proteinase K 200 μg/mL. The samples were incubated for 1 h at 56°C with frequent vortexing. DNA was extracted using phenol/chloroform methods and precipitated with NaCl 5 M in ethanol at −80°C. The DNA was resuspended in DNAse and RNAse free water and subjected to PCR using primers and conditions described elsewhere (Bibalan et al., 2014). PCR products were then electrophoresed in 2% agarose gel and visualized using Gel Doc EZ System Bio-Rad.

### Statistics

The data were analyzed using two-way analysis of variance (ANOVA) followed by Bonferroni multiple comparison *post hoc* test using GraphPad Prism (version 6.0). *P* values < 0.05 were considered statistically significant.

## Results

### Azan-7 Inhibits QS Signaling in MRSA

A recent study described Diflunisal’s anti-virulence activity against MRSA (Khodaverdian et al., 2013). Since its low solubility and reputation for adverse effects in humans has not led to a broad usage, we set out to synthesize 16 aza-derivatives with a similar chemical backbone (Carta et al., 2018) in the attempt to identify more soluble and less toxic compounds to inhibit *S. aureus* QS communication (Table 3).

**TABLE 3:**
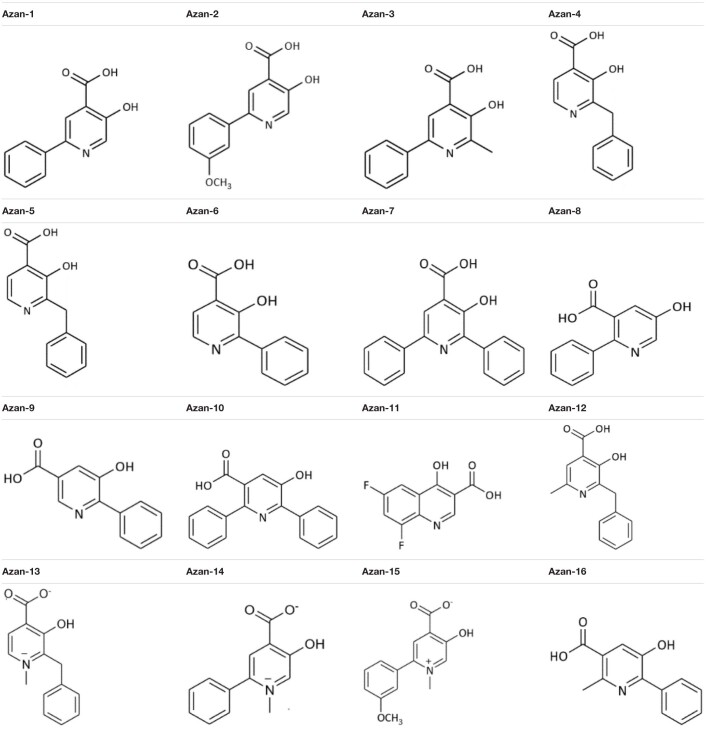
Aza-derivatives screened in the study.

**FIGURE 1:**
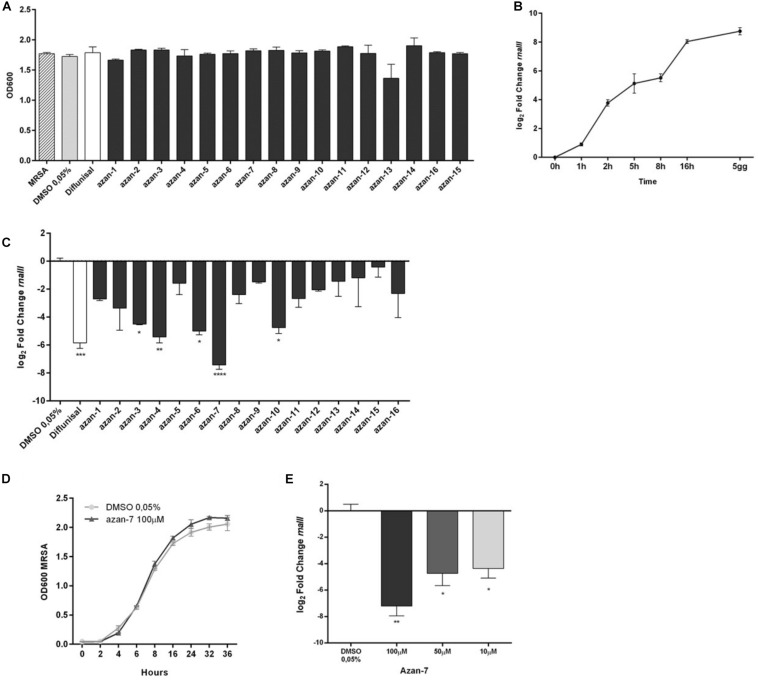
Screening of aza-analogs. **(A)** MRSA (10^6^ CFU/mL) were cultured in presence of Azan-derivatives (100 μM), Diflunisal (100 μM), or DMSO 0.05% (vehicle). Bacterial growth was evaluated 16 h later by measuring the optical density (OD) at 600 nm. Data are reported as mean ± SEM (*n* = 3). **(B)** MRSA (10^6^ CFU/mL) were cultured in LB and *rnaIII* mRNA transcripts were determined by qRT-PCR at the specified time points. Results are reported as log_2_ of relative gene expression (*n* = 3). **(C)** MRSA (10^6^ CFU/mL) were cultured in LB with Azan-derivatives (100 μM), Diflunisal (100 μM), or DMSO 0.05% for 16 h. *rnaIII* mRNA transcript levels were determined by qRT-PCR. Results are reported as log_2_ of relative gene expression (*n* = 3). ****P < 0.0001; ***P < 0.001; **P < 0.01; *P < 0.05 vs. MRSA treated with DMSO 0.05%. **(D)** MRSA (10^6^ CFU/mL) were cultured in presence of Azan-7 (100 μM) or 0.05% DMSO (vehicle). Bacterial growth was evaluated up to 16 h by measuring the optical density (OD) at 600 nm (*n* = 3). **(E)** MRSA (10^6^ CFU/mL) were cultured in LB with Azan-7 (100, 50, and 10 μM). *rnaIII* mRNA transcript levels were determined by qRT-PCR after 16 h. Results are reported as log_2_ of relative gene expression (*n* = 3). **P < 0.01; *P < 0.05 vs. MRSA treated with DMSO 0.05%. All data are reported as mean ± SEM.

To assess the aza-derivatives’ direct toxicity on bacteria and eukaryotic cells, we checked their effects on exponentially growing MRSA. The compounds showed no effect on the bacterial growth rate (Figure 1A).

Since the anti-virulence activity of QS inhibitors has been correlated to a reduction in *agr*-mediated expression, we screened the 16 small aza-derivatives’ ability to interfere with AIP signaling by quantifying the *rnaIII* mRNA level. We first assessed the time course of *rnaIII* expression in MRSA maintained in a planktonic culture over 120 h. *rnaIII* mRNA reached its highest expression following 16 h of culture, whereas we observed only a slight increase between 16 h and 5 days (Figure 1B). Therefore, we decided to perform all the experiments at 16 h of treatment. MRSA was cultured in the absence or presence of the 16 aza-derivatives, and 16 h later, total RNA was extracted to quantify *rnaIII* mRNA expression by qRT-PCR. While five molecules (Azan-3, Azan-4, Azan-6, Azan-7, and Azan-10) significantly downregulated the *rnaIII* mRNA level (Figure 1C), Azan-7 was the only compound that reduces *rnaIII* expression at higher extent as compare with Diflunisal. We, therefore, focused our experiment in Azan-7. We next performed dose-response analyses to identify the most effective dose to be used in our studies. When we measured mRNA levels of *rnaIII* on MRSA treated with different Azan-7 concentrations, we found that 100 μM was the most effective (Figure 1E). We excluded the direct toxicity effect of Azan-7 on exponentially growing MRSA (Figure 1D), and the IC_50_ values were higher than 2 mM on the tested eukaryotic cell lines (Supplementary Figure 1).

### Azan-7 Inhibits the Expression of Major Virulence Factors Regulated by the *agr*-System

**FIGURE 2:**
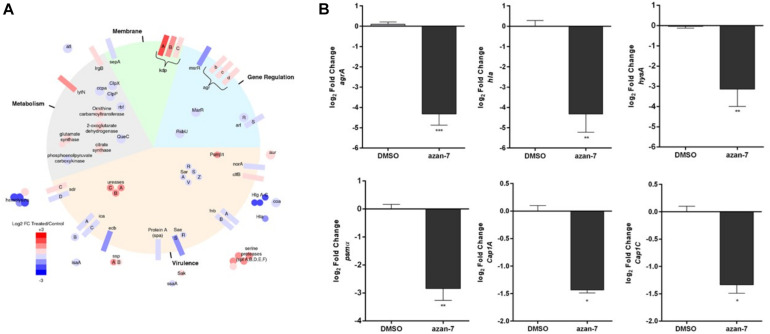
Effect of Azan-7 on *agr*-regulated gene transcription. **(A)** Graphic representation showing the category of microarray results in MRSA after 16 h of culture with Azan-7 (100 μM). All the major virulence factors are represented; the others are representative of the category. **(B)** qPCR was performed to assess mRNA transcript levels of *hla*, *psm*α, *cap1A*, *agrA*, *hysA*, and *cap1C* in MRSA treated with Azan-7 (100 μM) or DMSO 0.05% for 16 h. Results are reported as log_2_ of relative gene expression. The data are expressed as mean ± SEM, *n* = 3. ***P < 0.001; **P < 0.01; *P < 0.05 vs. MRSA treated with DMSO 0.05%.

We investigated the transcriptional impact of Azan-7 on *agr*-modulated virulence genes by comparing transcript levels obtained from MRSA vehicle-treated cultures with MRSA cultures treated with Azan-7. The obtained RNA-seq data have been deposited (GSE161844, release public date November 20, 2020) and are available at https://www.ncbi.nlm.nih.gov/geo/query/acc.cgi?acc=GSE161844. We selected *a priori* a set of genes known to be functionally related to *agr* QS system (Supplementary Table 1). We then confirmed our results using qRT-PCR. We did this by retrieving all the genes used to construct a Boolean network describing QS in *S. aureus* (Audretsch et al., 2013). Figure 2A illustrates Azan-7 activity of downregulation (blue) and upregulation (red). We classified the genes into four categories: virulence, metabolism, membrane, and gene expression. Azan-7 inhibited the expression of several genes involved in virulence; the downregulation of *hla*, *hlgA-C*, and hemolysins was particularly interesting. We also found that the compound decreased the transcript levels of several genes involved in biofilm formation, such as *atl* coding Atl enzymes, a major autolysin *S. aureus* surface protein formed by two domains, amidase and glucosaminidase (Porayath et al., 2018). Atl plays an essential role in biofilm formation, particularly in the early stage, as Atl promotes attachment to polystyrene surfaces and binding to host extracellular proteins. Moreover, Atl facilitates the cell lysis leading to the release of DNA in the extracellular compartment that is one of the crucial matrix molecules involved in biofilm formation (Bose et al., 2012). The treatment with Azan-7 also inhibited the expression of *Rbf* that represses *icaR* (negative regulator of *icaADBC*) transcription with a concomitant increase in *icaADBC* expression and formation of PNAG (poly-N-acetylglucosamine), biopolymer of the bacterial cell wall involved in biofilm production (Luong et al., 2009).

Data were confirmed by qRT-PCR for *agrA*, *hla*, *hysA*, *psm*α, *cap1C*, and *cap1A*, genes controlled by the *agr* system. Azan-7 significantly downregulated the expression of the tested genes (Figure 2B). The downregulation of gene transcripts specific for virulence factors was not due to bacteriostatic or bactericidal effects since Azan-7 did not impact bacterial growth and viability (Figure 1D).

**FIGURE 3:**
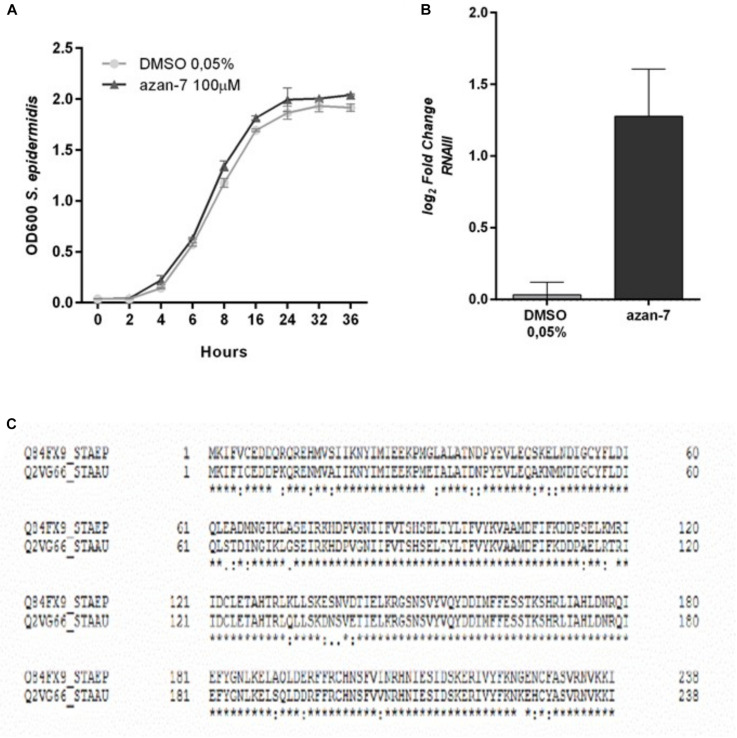
Lack of effect of Azan-7 in *S. epidermidis* cultures. **(A)** *S. epidermidis* (10^6^ CFU/mL) were cultured in presence of Azan-7 (100 μM) or 0.05% DMSO (vehicle). Bacterial growth was evaluated 16 h later by measuring the optical density (OD) at 600 nm. Data are reported as mean ± SEM (*n* = 3). **(B)** *S. epidermidis* (10^6^ CFU/mL) were treated with Azan-7 (100 μM) or DMSO 0.05% for 16 h. *rnaIII* mRNA transcript levels were determined by qRT-PCR. Results are reported as log2 of relative gene expression. Data are reported as mean ± SEM (*n* = 3). **(C)** Comparison of AgrA protein aminoacidic sequence between *S. epidermidis* (first line) and MRSA (second line). AgrA proteins show homology of 87%. * = identity; . = semi-conservative amino acid; : = conservative amino acid; gap = non conservative amino acid (UniProt).

To verify if Azan-7 was active against other Gram-positive bacteria, we evaluated its effect on *S. epidermidis* growth and *rnaIII* mRNA expression. As shown in Figure 3, Azan-7 interfered neither with *S. epidermidis* growth (Figure 3A) nor with *rnaIII* expression (Figure 3B), even though the homology of AgrA proteins of *S. aureus* and *S. epidermidis* is about 80% (Figure 3C).

### Azan-7 Affects the Transcriptional Function of AgrA

**FIGURE 4:**
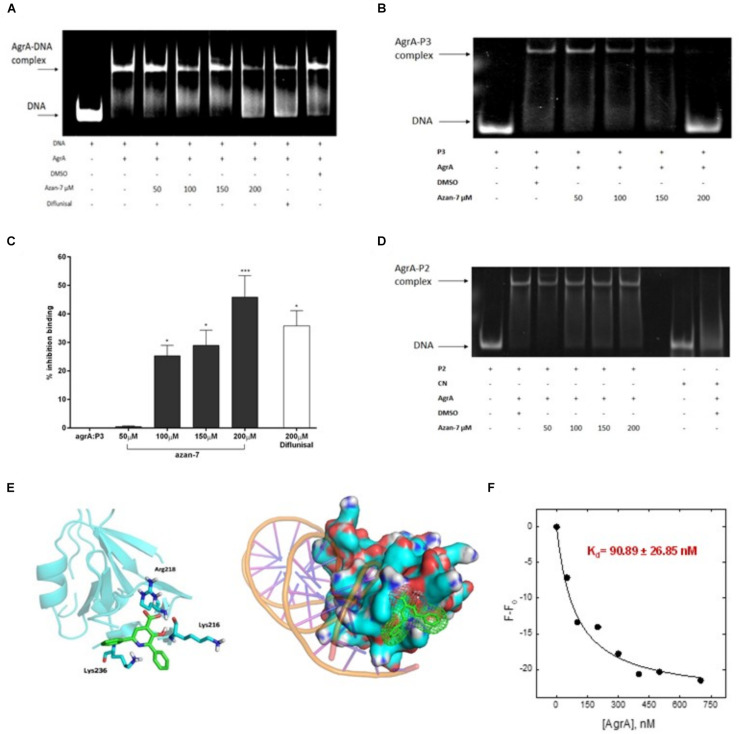
Azan-7 binds to AgrA and inhibitsDNA complex formation. **(A,B)** Recombinant AgrA was mixed withincreasing concentrations of Azan-7 (50–200 μM), Diflunisal (200 μM), or vehicle (DMSO 0.05%) and incubated with FAM-labeled P3 oligonucleotide. Samples were analyzed by gel shift assay (EMSA) and separated on 10% native polyacrylamide gel and formation of AgrA:P3 complexes was visualized by GE Typhoon FLA 2000 PhosphorImager. **(C)** The percentage of inhibition of DNA/AgrA binding for Azan-7 was quantified by densitometry analysis. Data are reported as mean ± SEM and report values of three independent experiments. ***P < 0.01; *P < 0.05 vs. AgrA:P3. **(D)** Recombinant AgrA was mixed with increasing concentrations of Azan-7 (50–200 μM), or vehicle (DMSO 0.05%) and incubated with FAM-labeled P2 oligonucleotide. AgrA:P2 complex formation was evaluated as described in panels **(A,B)**. **(E)** *In silico* docking of Azan-7 to AgrA of MRSA. Azan-7 binds three amino acids: Arg218, Lys216, and Lys236. **(F)** Azan-7 (200 nM, 0.5 ml) in TBS, pH 8.0 and increasing concentrations of AgrA (0–700 nM) were excited at 342 nm after incubation at 37°C for 15 min. The decrease of fluorescence intensity was recorded at 475 nm. The experimental data were reported as F–F_0_, where F is the fluorescence intensity at each AgrA concentration and F_0_ is AgrA fluorescence in the absence of Azan-7 substrate. The relative equilibrium dissociation constant was K_d_ = 90.89 ± 26.85 nM.

To better understand Azan-7 mechanism of action, we used EMSA to evaluate the ability of Azan-7 to interfere with AgrA’s binding to oligonucleotide sequences corresponding to P2 promoters, increasing production of AIP, and P3 promoter, driving the expression of several virulence factors. P2-AgrA binding was not significantly affected by Azan-7 (Figure 4D), whereas the AgrA-P3 complex formation was significantly reduced in the presence of Azan-7, indicating inhibition of AgrA binding to P3 (Figures 4A,B). The percentage of inhibition binding for Azan-7 was quantified by densitometry analysis (Figure 4C). Overall, our data suggest that Azan-7 inhibits the regulator transcription factor by binding to P3, thus reducing virulence factors.

### Azan-7 Interacts With the Transcriptional Function of AgrA

To propose a potential binding mode for Azan-7 in AgrA, docking simulations were conducted on the binding site for small molecules previously described by P. Leonard (Leonard et al., 2012). An excellent steric complementary between target and ligand was observed. Moreover, we expected to see that the carboxylate function of Azan-7 formed a salt bridge with the Arg218 side chain, while the hydroxyl function formed an H-bond with the amide C = O of Lys216. By re-running the docking experiment considering the Lys216 sidechain as flexible, we also observed the potential formation of two other interactions: one hydrogen bond between pyrimidine nitrogen and protonated amine sidechain as well as an arene-cation (Figure 4E), also showing an excellent electrostatic complementarity. It would seem then that Azan-7 interferes with AgrA/DNA binding by preventing an electrostatic interaction between protein AgrA amino acids (Arg218, Lys216, and Lys236) and DNA (Sidote et al., 2008).

AgrA/Azan-7 K_d_ was evaluated by Fluorescence Spectroscopy(Figure 4F). Samples (200 nM, 1.5 ml) of Azan-7 at increasing concentrations of AgrA (0–700 nM) were excited at 342 nm after incubation at 37°C for 15 min. The decrease of fluorescence intensity was recorded at 475 nm. The experimental data were reported as F-F_0_, where F is the fluorescence intensity at each AgrA concentration and F_0_ is AgrA fluorescence in the absence of Azan-7 substrate. After data fitting with eq. 1, the relative equilibrium dissociation constant was K_d_ = 90.89 ± 26.85 nM.

### Azan-7 Inhibits Major MRSA Virulence Factors

**FIGURE 5:**
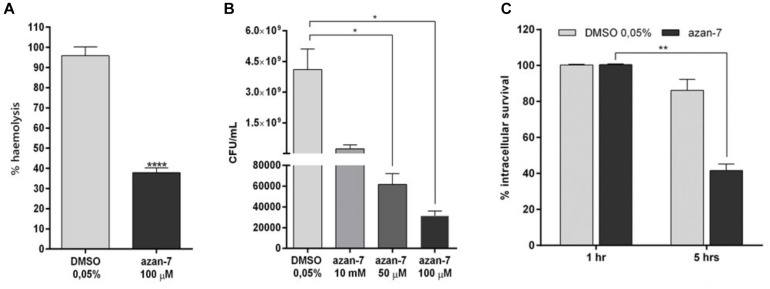
Azan-7 reduces alpha-haemolysin production, resistance to low pH, and survival to macrophage killing. **(A)** MRSA (10^6^ CFU/mL) were cultured with Azan-7 (100 μM) or vehicle (DMSO 0.05%) for 16 h, and incubated for 1 h with rabbit erythrocytes. Erythrocytes lysis was evaluated measuring optical absorbance at 541 nm. Haemolysis produced from MRSA cultured with DMSO was arbitrarily assigned 100% haemolysis (*n* = 3). ****P < 0.0001 vs. MRSA treated with DMSO 0.05%. **(B)** MRSA (10^6^ CFU/mL) were cultured in presence of Azan-7 (100, 50, or 10 μM) or vehicle (DMSO 0.05%) for 16 h and then incubated at pH 2.5 for 2 h. Bacterial survival was quantified by seeding bacteria on LB agar plates for vital count. Live bacteria obtained from culture with DMSO were fixed at 100% survival (*n* = 3). *P < 0.05 vs. MRSA cultured with DMSO 0.05%. **(C)** MRSA (10^6^ CFU/mL) were cultured in the presence of Azan-7 (100 μM) or vehicle (DMSO 0.05%) for 16 h and then were co-incubated with murine macrophages (RAW264 cells) to assess phagocytic activity for 1 or 5 h. Viable intracellular bacteria were quantified by seeding disrupted macrophages on LB agar plates for vital count. Data are reported as the percentage of viable bacteria after 5 h (*n* = 4) over the viable bacteria after 1 h, set at 100% of survival for the respective treatment. The data are reported as the mean ± SEM. **P < 0.01 vs. MRSA cultured with DMSO 0.05%.

To evaluate Azan-7 ability to inhibit the production of alpha-haemolysin from the *agr* regulon, we quantified the haemolysis activity present in MRSA culture toward rabbit erythrocytes. The amount of hemoglobin released from the lysed red blood cells, measured using spectrophotometric analysis, was significantly reduced in MRSA grown for 16 h in the presence of Azan-7 (Figure 5A), in agreement with the reduced expression of the *hla* gene (Figure 2A).

Since *agr* regulates transcripts involved in acid resistance, we next investigated Azan-7 capacity to affect the ability of MRSA to survive the killing in macrophage phagolysosomes. We compared the survival of MRSA with or without exposure to Azan-7 by measuring the survival of MRSA in macrophage or low pH medium. Azan-7 markedly reduced MRSA survival in macrophages and at low pH compared with bacteria exposed to vehicle (Figures 5B,C).

### MRSA Does Not Acquire Resistance to Azan-7

**FIGURE 6:**
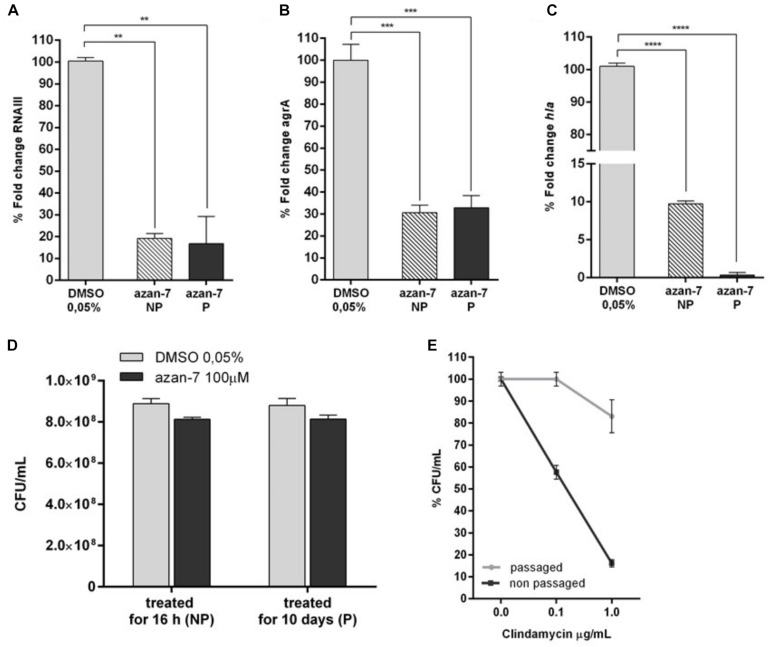
Long term exposure to Azan-7 fails to select resistant strains. MRSA (10^6^ CFU/mL) were treated for 16 h with Azan-7 (100 μM) or DMSO 0.05% (NP = non-passaged). Cells were collected and RNA was extracted. In a different set of experiments, MRSA (10^6^ CFU/mL) were treated every day for 10 days with Azan-7 (100 μM) or DMSO 0.05% (P = passaged). RNA was extracted and mRNA transcript levels specific for *rnaIII* **(A)**, *agrA* **(B)**, and *hla* **(C)** were quantified by qRT-PCR. Data are reported as the mean ± SEM (*n* = 3). **P < 0.01, ***P < 0.001, ****P < 0.0001 vs. MRSA cultured with DMSO 0.05%. **(D)** MRSA cultures were treated for 16 h with Azan-7 (100 μM) or DMSO 0.05% (NP = non-passaged) and with Azan-7 (100 μM) or DMSO 0.05% every day for 10 days (P = passaged). At the end of incubations, bacterial cultures were diluted and plated on LB agar for CFU enumeration. Data are reported as the mean ± SEM (*n* = 4). **(E)** MRSA were treated for 16 h (NP) or every day for 10 days with clindamycin 0.25 μg/mL (P). At the end of incubations, bacterial cultures were diluted and plated on LB agar for CFU enumeration. Data are reported as percent fold change of bacterial colonies calculated over MRSA treated with DMSO (*n* = 6).

All antibiotics exert selective pressure on bacteria, a feature that promotes the acquisition of resistance. Undeniably, *S. aureus* very rapidly develops resistance to new antimicrobial drugs. To verify if MRSA develops resistance to Azan-7, we treated MRSA planktonic culture with Azan-7 for 10 days and then evaluated the persistence of the effects by quantifying *rnaIII*, *agrA*, and *hla* transcript levels. As shown in Figure 6, MRSA treated with Azan-7 reported downregulation of the target genes at the same extent at 1 and 10 days of treatment (Figures 6A–C), suggesting that no resistance had developed. Results were confirmed by CFU enumeration in MRSA cultures with Azan-7 for 16 h or 10 days (Figure 6D). On the contrary, bacteria cultured for 10 days with clindamycin did acquire resistance to the antibiotic (Figure 6E).

### Azan-7 Potentiates Clindamycin’s Effects on MRSA

**FIGURE 7:**
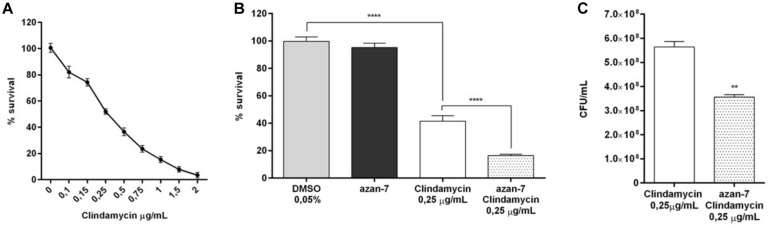
Azan-7 enhances the susceptibility to clindamycin in planktonic cultures of MRSA. **(A)** Determination of clindamycin MIC_50_ on planktonic MRSA cultures (*n* = 3). **(B)** Planktonic MRSA cultures were treated with vehicle (DMSO 0.05%), clindamycin (0.25 μg/mL), Azan-7 (100 μM), or clindamycin plus Azan-7. The bacterial cells were quantified by measuring OD_600 nm_ and data are reported as % changes over the control (*n* = 3). ****P < 0.0001 vs. MRSA treated with DMSO 0.05%, ****P < 0.0001 vs. MRSA treated with clindamycin. **(C)** Alternatively, MRSA cultures were diluted and plated on LB agar for colony enumeration. Data are reported as mean ± SEM (*n* = 3). **P < 0.01 vs. MRSA treated with clindamycin 0.25 μg/mL).

**FIGURE 8:**
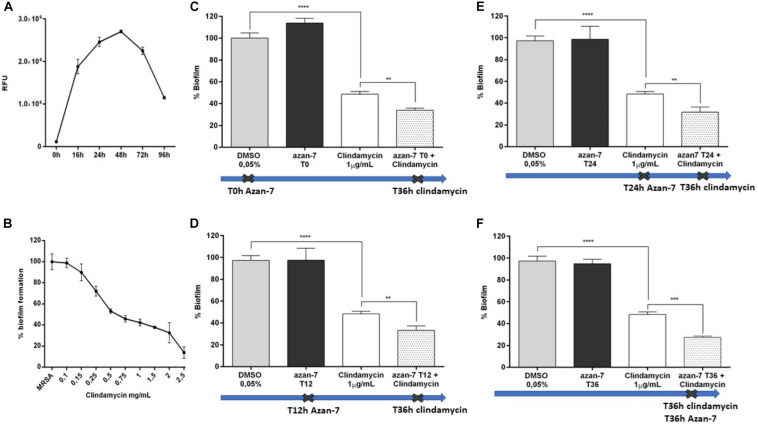
Azan-7 enhances the susceptibility of MRSA biofilm to clindamycin. **(A)** MRSA culture was seeded in 96-well and incubated at 37°C in static conditions. Adhering cells were determined at the specified time points after incubation at 37°C for 20 min with resazurin 0.01%. Biofilm formation was evaluated by measuring the relative fluorescence units (RFU) using a fluorimeter (Ex = 530–570 nm, Em = 590–620 nm). Data are reported as mean ± SEM (*n* = 3). **(B)** To determine MIC_50_ value, MRSA biofilms were incubated with clindamycin at different concentrations. Biofilms were evaluated as described above. Data are reported as mean ± SEM (*n* = 3). **(C–F)** Percentage of residual MRSA biofilm after treatment with clindamycin alone, Azan-7 added at different times alone or in combination with clindamycin. Azan-7 (100 μM) was added at 0 **(C)**, 12 **(D)**, 24 **(E)**, or 36 h **(F)**, as indicated by the crosses. Clindamycin was always added at 36 h. Data are reported as mean ± SEM (*n* = 3). ****P < 0.0001 vs. biofilm treated with DMSO 0.05%, **P < 0.01 vs. biofilm treated with clindamycin alone.

To evaluate if Azan-7 could enhance antibiotics activity, we quantified the bacterial survival after overnight incubation with the compound in the presence or absence of clindamycin, an antibiotic mostly used to treat invasive community-acquired MRSA skin and soft tissue infections (Liu et al., 2011). We first determined MIC_50_ value of clindamycin in MRSA isolates both in planktonic cultures and biofilm as it is known that bacteria attached to a surface and growing as a biofilm are more resistant to antibiotics (Davies, 2003). As expected, the MIC_50_ value of clindamycin was higher in MRSA biofilm than in planktonic cultures (1 vs. 0.25 μg/mL, respectively; Figures 7A, 8B). Spectrophotometric (Figure 7B) and vital counts (CFU/mL) analysis (Figure 7C) showed that clindamycin antimicrobial activity significantly increased in the presence of Azan-7 in planktonic cultures. In addition, Azan-7 and clindamycin have synergistic activity as determined by chekerboard assay that reported the Fractional Inhibitory Concentration (FIC) index corresponding 0.45.

We next tested Azan-7 in the presence and absence of antibiotics on biofilm since it has been reported that device-associated infections due to biofilm formation correlates with antibiotic treatment failure (Günther et al., 2017). We observed the highest biofilm production after 48 h of growth (Figure 8A). The effects of Azan-7 plus clindamycin were then tested following 48 h of incubation. While Azan-7 *per se* had no relevant effects on *S. aureus* biofilm formation, it significantly enhanced the clindamycin’s effects on biofilm when Azan-7 was added to the antibiotic at different time points following biofilm development (Figures 8C–F).

### Azan-7 Inhibits the Expression of Virulence Factors in Clinical Isolates Carrying Different *agr* Types

**FIGURE 9:**
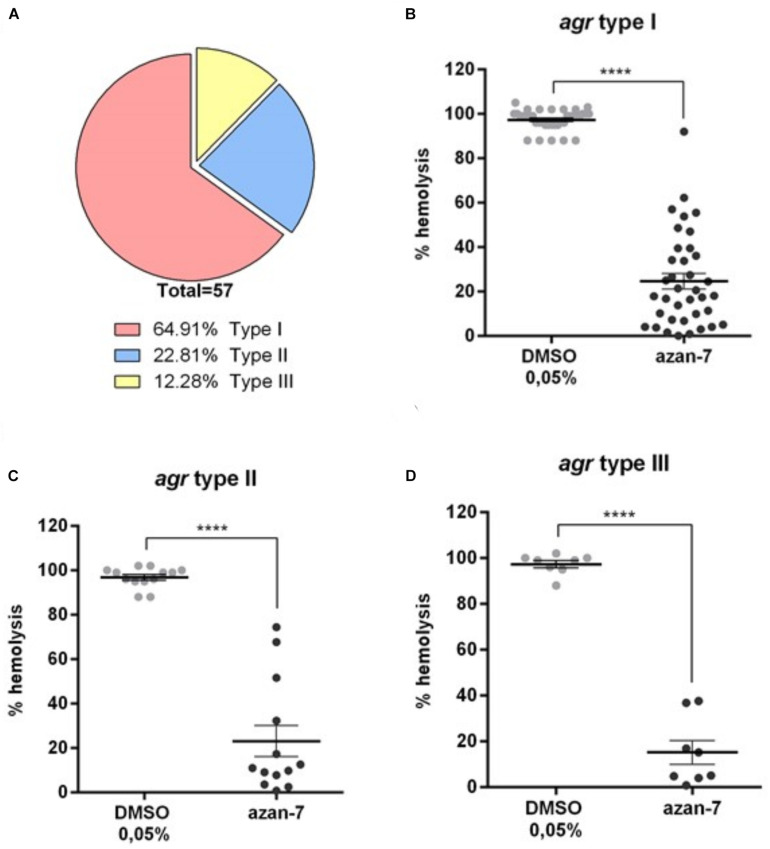
Azan-7 inhibits α-haemolysin production in *S. aureus* clinical isolates. **(A)** Fifty-seven *S. aureus* clinical isolates were characterized for the agr types by PCR. **(B–D)** *S. aureus* agr-type I, II, and III were cultured in the presence of Azan-7 (100 μM) or vehicle (DMSO 0.05%) for 16 h. α-haemolysin production was evaluated measuring rabbit erythrocytes lysis. Haemolysis obtained from strains treated with DMSO was arbitrarily assigned 100% haemolysis. Data are reported as mean ± SEM (*n* = 3). ****P < 0.0001 vs. the same strains treated with DMSO 0,05%.

As four different *agr S. aureus* types have been described so far, we characterized the *agr* types in 57 clinical isolates to evaluate the anti-virulence activity of Azan-7 on genotypically different clinical strains. As shown in Figure 9A, most of the collected clinical isolates below to the *agr* type I. Nevertheless, we classified the clinical isolates by the *agr* type, and we assessed the anti-virulence activity of Azan-7 using the haemolysis assay. Azan-7 significantly inhibited MRSA-induced haemolysis in all the tested clinical isolates, independently from the *agr* types (Figures 9B–D). However, the strongest haemolysis reduction (∼80%) was observed in clinical isolates of the *agr* type III (Figure 9D).

## Discussion

The *agr* controls one of the most critical operons in *S. aureus* biology as it regulates virulence gene expression. It has been demonstrated that Diflunisal possesses an anti-virulence activity against MRSA, probably inhibiting AgrA protein binding to promoter P3 (Khodaverdian et al., 2013). When we screened a library of 16 small aza-derivatives (Table 3) in the effort to identify more active, less toxic molecules, we found that Azan-7 showed remarkable anti-virulence activities since it significantly reduced the activation of all agr-types clinical isolates (Figure 9) in a non-biocidal manner (Figure 1). *Agr* deregulation was first demonstrated via RNA-seq analysis, showing that Azan-7 affected a variety of *agr*-regulated transcripts (Supplementary Figure 2 and Supplementary Table 1). Indeed, docking analysis showed that Azan-7 fits in the AgrA active domain and interacts with the Arg218, Lys216, and Lys236 amino acid residues (Figure 4E). As this domain is involved in DNA binding, it is responsible for AgrA-mediated QS signaling (Leonard et al., 2012). The molecule seems to belong to the same category of AgrA inhibitors as savarin, the small molecule inhibitor (Sully et al., 2014) or ω-Hydroxyemodin (Daly et al., 2015), but Azan-7 exhibits a lower K_d_ for AgrA (90.89 nM, Figure 4F). Moreover, Azan-7 showed a high specificity for Agr MRSA since it did not suppress *rnaIII* expression in *S. epidermidis* despite the 80% homology between the proteins in these two related bacterial species (Figure 3C). The fact that the molecule does not affect the commensal bacteria suggests that it does not interfere with the resident microbiota (Cogen et al., 2008).

The unique virulence of MRSA is due to the production of a large number of toxins and peptides which target host cell membranes (Vandenesch et al., 2012). The expression of the toxin alpha-haemolysin (Hla), one of the best-known toxins produced by *S. aureus*, is a cytolytic toxin that forms pores in host cell membranes causing cell lysis (Kong et al., 2016). The expression of the toxin is indirectly upregulated by phosphorylated AgrA (Christian, 2018). Azan-7 has markedly reduced *hla* mRNA expression and MRSA haemolytic activity, both in laboratory strains (Figure 5A) and in clinical isolates carrying different *agr* type alleles (Figure 9). In *S. aureus*, the four *agr* types are characterized by different *agr* loci, and the main differences have been identified on two genes: *agrD*, which is responsible for encoding AIPs that differ from the variants (Bibalan et al., 2014), and *agrC*, which encodes the AgrC receptor protein in which amino acid variation conferring signal specificity has been observed (Choudhary et al., 2018). Type I and type II *S. aureus* are known for their clinical relevance and are commonly associated, respectively, with invasive (Bibalan et al., 2014) and nosocomial infections (Parlet et al., 2019). Despite the *agr* locus variations, the *agrA* gene did not show any difference between the four types, and the protein AgrA is member of a family of conserved response regulators (Koenig et al., 2004). These observations support our evidence showing that Azan-7 binds to AgrA (Figure 4) to inhibit its activity.

Biofilm formation is another important virulence factor characterizing MRSA infections that are usually more virulent, resistant to therapy, and tend to become chronic (Costerton et al., 1999). Previous studies have produced conflicting results showing that AgrA-binding molecules enhance or inhibit biofilm formation (Costerton et al., 1999; Mootz et al., 2016). Although Azan-7 shows no anti-biofilm effect, it enhances antibiotic activity against biofilm when the compound is administered with clindamycin (Figure 8). Azan-7 did not inhibit the production of proteases involved in biofilm dispersion, thus preventing colonization at new infection sites (Costerton et al., 1999; Baldry et al., 2016) and reduced the expression of genes involved in capsular protein synthesis (Supplementary Table 1). Hypothetically, even if Azan-7 appears self-defeating by inducing AgrA inhibition, by favoring continuous protease production and biofilm dispersion, biofilm’s structure is weakened, rendering it more permeable and susceptible to the effect of the antibiotic.

Some of the virulence factors regulated by the Agr-QS system include the ability to escape macrophage killing by managing to survive in an acidic setting and the production of phenol-soluble modulins (PSMs), a family of small proteins that MRSA uses for phagosomal escape (Münzenmayer et al., 2016). Our findings have demonstrated that Azan-7 markedly reduces the ability of MRSA to survive in acidic environments and to tolerate conditions that mimic the phagolysosomal environment. By inhibiting QS signaling, Azan-7 blocks bacteria defense mechanisms and inhibits bacteria’s intracellular survival, indirectly favoring host immune system activity.

The utility of any new antimicrobial agent can be jeopardized by bacteria’ ability to rapidly develop resistance to the new drug. It is well known that *S. aureus* has become resistant to all the antibiotics to which it has been exposed. This feature is linked to the antibiotic’s mechanism of action and is ingrained in the selective pressure on bacteria to promote the emergence of resistant strains. As opposed to classical antibiotics, Azan-7 does not interfere with bacteria survival or growth (Figure 1), nor does it induce resistant strains (Figures 7, 8).

## Conclusion

In conclusion, Azan-7 is a QS inhibitor, anti-virulence compound belonging to a new category of anti-microbial agents that utilizes complementary mechanisms of actions. Azan-7 (i) reduces *S. aureus*’ ability to express toxins capable of compromising host tissues’ physiology; (ii) interferes with the resistance of the bacteria to host defense mechanisms; (iii) acts together with the antibiotic to disrupt biofilm formation. Our findings lead to the conclusion that Azan-7 is endowed with important therapeutic effects and could be proposed in combination with traditional antibiotics to treat *S. aureus* induced nosocomial-acquired biofilm-based infections. The administration of Azan-7 can lower the therapeutic doses of antibiotics, thus preventing antibiotic resistance in patients.

## Data Availability Statement

The RNA seq data has been uploaded to the GEO–GSE161844. Other data can be accessed with the accession numbers GSM4915807-GSM4915810.

## Author Contributions

GB, PB, and IC designed the research, performed the experiments, analyzed the data, and wrote the manuscript. VR, AS, and AP performed the microbiological experiments. IA and VDF performed fluorescence binding experiments. FS collected and characterized the clinical isolates. MD, GM, and MF synthesized the compounds. MF performed computational studies. All authors listed approved the manuscript for publication.

## Conflict of Interest

The authors declare that the research was conducted in the absence of any commercial or financial relationships that could be construed as a potential conflict of interest.

## Footnotes

1 Marvin, *Version 5.5.0.1, Program B*. Budapest: ChemAxon

2 https://www.uniprot.org

## Supplementary Material

Supplementary Figure 1 Azan-7 did not report cytotoxicity on eukaryotic cells. A549 and Caco2 human cell lines were cultured with Azan-7 at concentrations ranging from 0 to 2 mM for 72 h. Incubation with DMSO 0.05% served as control (0 mM). Cell viability was assessed by MTT (3-(4,5-dimethylthiazol-2-yL)-2,5- diphenyltetrazolium bromide) assay. Data (*n* = 3) are reported as percent cell viability calculated over the control.

Supplementary Table 1 RNA sequencing data.
