# Supplementary figures and images for: A Novel Aza-Derivative Inhibits agr Quorum Sensing Signaling and Synergizes Methicillin-Resistant Staphylococcus aureus to Clindamycin
Source: Front Microbiol. 2021 Feb 9;12:610859. doi: 10.3389/fmicb.2021.610859 (PMC7899991; doi:10.3389/fmicb.2021.610859)

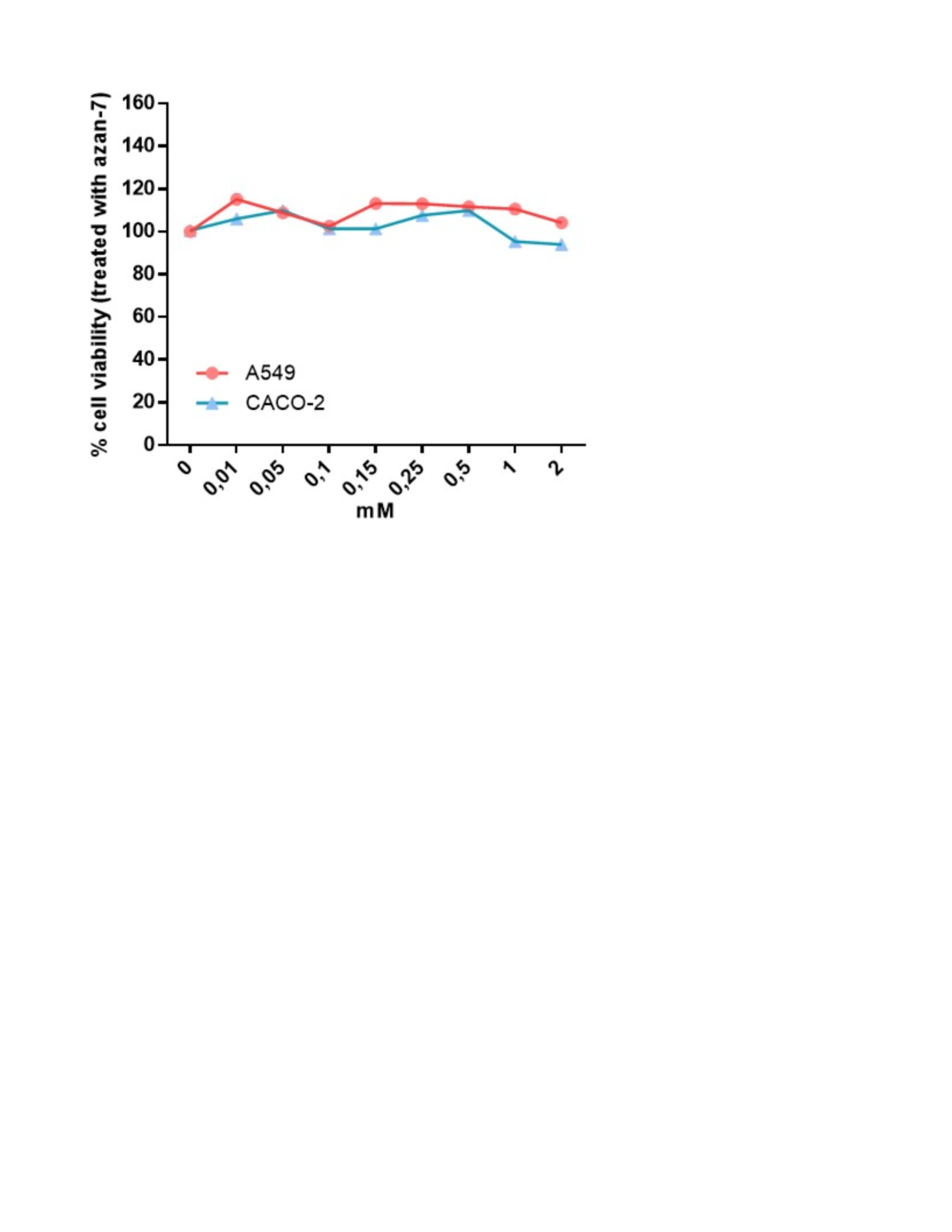

Supplement: Supplementary Figure 1 — Azan-7 did not report cytotoxicity on eukaryotic cells. A549 and Caco2 human cell lines were cultured with Azan-7 at concentrations ranging from 0 to 2 mM for 72 h. Incubation with DMSO 0.05% served as control (0 mM). Cell viability was assessed by MTT (3-(4,5-dimethylthiazol-2-yL)-2,5- diphenyltetrazolium bromide) assay. Data (n = 3) are reported as percent cell viability calculated over the control. [file Image_1.TIF]
