# Supplementary material for: A Novel Aza-Derivative Inhibits agr Quorum Sensing Signaling and Synergizes Methicillin-Resistant Staphylococcus aureus to Clindamycin
Source: Front Microbiol. 2021 Feb 9;12:610859. doi: 10.3389/fmicb.2021.610859 (PMC7899991; doi:10.3389/fmicb.2021.610859)
Supplement: Supplementary Table 1 — RNA sequencing data. [file Table_1.PDF]

Table 1 Supplementary Material. Gene expression as obtained by RNA sequencing analysis. Comparisons were made between MRSA vehicle (DMSO 0,05%) and MRSA treated with Azan-7 (100 µM). In red are reported the genes whose expression was increased by the treatment. In blue the genes with the reduced expression.

Gene ID	Gene symbol	Blast gene description	Vehicle *vs* Azan-7 (log2 FoldChange)
SAUSA300_RS11715	sepA	Multidrug resistance efflux pump SepA	-0,514004551
SAUSA300_RS11190	kdpA	Potassium-transporting ATPase potassium-binding subunit;Potassium-transporting ATPase potassium-binding subunit 1	2,588374583
SAUSA300_RS11185	kdpB	Potassium-transporting ATPase ATP-binding subunit 2;Potassium-transporting ATPase ATP-binding subunit	1,060953053
SAUSA300_RS11180	kdpC	Potassium-transporting ATPase KdpC subunit	0,894011994
SAUSA300_RS13600	-	HTH-type transcriptional regulator PchR;Transcriptional regulator ZitR;Uncharacterized HTH-type transcriptional regulator YvnA	1,521911571
SAUSA300_RS14145	-	Putative HTH-type transcriptional regulator YvaV;HTH-type transcriptional repressor OpcR;HTH-type transcriptional repressor GbsR	-0,640738058
SAUSA300_RS13640	-	Uncharacterized HTH-type transcriptional regulator YkoM;HTH-type transcriptional regulator MhqR;Uncharacterized HTH-type transcriptional regulator YpoP	-0,716450337
SAUSA300_RS03605	mgrA	Organic hydroperoxide resistance transcriptional regulator;HTH-type transcriptional regulator MgrA;HTH-type transcriptional regulator SarZ	-0,227130876
SAUSA300_RS14260	-	HTH-type transcriptional regulator PetP	-0,35702089
SAUSA300_RS03740	queC	7-cyano-7-deazaguanine synthase	-0,634870915
SAUSA300_RS05755	argF	Ornithine carbamoyltransferase	0,801342337
SAUSA300_RS07995	bmfBB	Lipoamide acyltransferase component of branched-chain alpha-keto acid dehydrogenase complex;Dihydrolipoyllysine-residue acetyltransferase component of pyruvate dehydrogenase complex	0,347464356
SAUSA300_RS07105	sucA	2-oxoglutarate dehydrogenase E1 component	0,238872273
SAUSA300_RS07100	sucB	Dihydrolipoyllysine-residue succinyltransferase component of 2-oxoglutarate dehydrogenase complex	0,158583259

SAUSA300_RS02380	gltB	Ferredoxin-dependent glutamate synthase 1;Glutamate synthase [NADPH] large chain	0,415970707
SAUSA300_RS02385	gltD	Glutamate synthase [NADPH] small chain;Putative glutamate synthase [NADPH];Glutamate synthase 2 [NADH], chloroplastic	0,629655619
SAUSA300_RS09470	pckA	Phosphoenolpyruvate carboxykinase (ATP)	-0,392173651
SAUSA300_RS08955	citZ	Citrate synthase;Citrate synthase 2	0,466207326
SAUSA300_RS09615	splB	Serine protease SplB	1,976654683
SAUSA300_RS09595	splF	Serine protease SplF	1,735846415
SAUSA300_RS09605	splD	Serine protease SplD;Serine protease SplF	1,342180013
SAUSA300_RS09620	splA	Serine protease SplA	1,188729918
SAUSA300_RS09600	splE	Serine protease SplE	0,935550099
SAUSA300_RS12365	ureC	Urease subunit alpha	1,538042866
SAUSA300_RS12360	ureB	Urease subunit beta	1,742431966
SAUSA300_RS12355	ureA	Urease subunit gamma	1,550604529
SAUSA300_RS00810	capB	Putative tyrosine-protein kinase CapB;Putative tyrosine-protein kinase YveL;Tyrosine-protein kinase YwqD	-0,954976312
SAUSA300_RS00815	capC	Putative tyrosine-protein phosphatase CapC;Tyrosine-protein phosphatase YwqE;Tyrosine-protein phosphatase CpsB	-0,737385123
SAUSA300_RS13080	hlgB	Gamma-hemolysin component B;Leukocidin-F subunit	-2,000472067
SAUSA300_RS13075	hlgC	Gamma-hemolysin component C	-2,089675739
SAUSA300_RS13070	hlgA	Gamma-hemolysin component A;Leucotoxin LukEv	-2,256158289
SAUSA300_RS05720	hla	Alpha-hemolysin;Gamma-hemolysin component B	-1,407649003
SAUSA300_RS05790	psmβ1		1,349896833
SAUSA300_RS05105	sspB	Staphopain B	0,469808085
SAUSA300_RS00585	spa	Immunoglobulin G-binding protein A	-0,390501615
SAUSA300_RS02920	sdrD	Serine-aspartate repeat-containing protein D	-0,317296059
SAUSA300_RS13525	fnbB	Fibronectin-binding protein A	-0,835651175
SAUSA300_RS13530	fnbA	Fibronectin-binding protein A	-0,47500723
SAUSA300_RS05670	ecb		-1,940170762
SAUSA300_RS05695	scc	Staphylococcal complement inhibitor	-1,771739692
SAUSA300_RS05690	efb	Fibrinogen-binding protein	-1,871451972
SAUSA300_RS10935	agrB	Accessory gene regulator protein B	0,545923601
SAUSA300_RS10945	agrC		0,496019379
SAUSA300_RS10940	agrD		0,255569018
SAUSA300_RS07115	arlR	Response regulator ArlR	-0,520606384
SAUSA300_RS07110	arlS	Signal transduction histidine-protein kinase ArlS	-0,401234303
SAUSA300_RS13915	isaA	Probable transglycosylase IsaA	-0,414811746

SAUSA300_RS06830	msrR	Regulatory protein MsrR;Polyisoprenyl-teichoic acid--peptidoglycan teichoic acid transferase TagU	-1,230525361
SAUSA300_RS11135	rsbU	Phosphoserine phosphatase RsbU	-0,350126151
SAUSA300_RS05135	atl	Bifunctional autolysin	-0,147678256
SAUSA300_RS09185	ccpA	Catabolite control protein A	-0,509346394
SAUSA300_RS04060	clpP	ATP-dependent Clp protease proteolytic subunit	-0,439691661
SAUSA300_RS08835	clpX	ATP-dependent Clp protease ATP-binding subunit ClpX	-0,515023364
SAUSA300_RS01370	lrgB	Antiholin-like protein LrgB	0,245268496
SAUSA300_RS06170	lytN	Probable cell wall hydrolase LytN	1,253437741
SAUSA300_RS06295	rbfA	Ribosome-binding factor A	-0,609021326
SAUSA300_RS14310	aur	Extracellular elastase;Zinc metalloproteinase aureolysin	0,640521456
SAUSA300_RS14270	clfB	Clumping factor B	0,279989092
SAUSA300_RS01180	coa	Staphylocoagulase	-0,514650507
SAUSA300_RS13530	fnbA	Fibronectin-binding protein A	-0,47500723
SAUSA300_RS13525	fnbB	Fibronectin-binding protein A	-0,835651175
SAUSA300_RS05720	hla	Alpha-hemolysin;Gamma-hemolysin component B	-1,407649003
SAUSA300_RS13080	hlgB	Gamma-hemolysin component B;Leukocidin-F subunit	-2,000472067
SAUSA300_RS13075	hlgC	Gamma-hemolysin component C	-2,089675739
SAUSA300_RS13070	hlgA	Gamma-hemolysin component A;Leucotoxin LukEv	-2,256158289
SAUSA300_RS10930	hld		0,399287655
SAUSA300_RS14455	icaA	Poly-beta-1,6-N-acetyl-D-glucosamine synthase	-0,467718818
SAUSA300_RS14470	icaC	Probable poly-beta-1,6-N-acetyl-D-glucosamine export protein	-0,587751744
SAUSA300_RS14465	icaB	Poly-beta-1,6-N-acetyl-D-glucosamine N-deacetylase	-0,441178907
SAUSA300_RS03650	norA	Quinolone resistance protein NorA	-0,210850132
SAUSA300_RS03710	saeR	Transcriptional regulatory protein ResD;Response regulator SaeR	-0,957417537
SAUSA300_RS03705	saeS	Histidine protein kinase SaeS	-1,200592278
SAUSA300_RS10540	sak	Staphylokinase	0,459811889
SAUSA300_RS00590	sarS	HTH-type transcriptional regulator SarU;HTH-type transcriptional regulator SarS	-0,602107643
SAUSA300_RS12390	sarR	HTH-type transcriptional regulator SarR	-0,408604158
SAUSA300_RS12880	sarZ	HTH-type transcriptional regulator SarZ	-0,469389472
SAUSA300_RS03250	sarA	Transcriptional regulator SarA;HTH-type transcriptional regulator SarT	-0,135582813
SAUSA300_RS12240	sarV	HTH-type transcriptional regulator SarV	-0,200096795
SAUSA300_RS02915	sdrC	Serine-aspartate repeat-containing protein C	0,259172431

SAUSA300_RS12415	ssaA	Staphylococcal secretory antigen ssaA2	-0,339428073
SAUSA300_RS05110	sspA	Glutamyl endopeptidase	1,157287185
SAUSA300_RS05105	sspB	Staphopain B	0,469808085
